# Supplementary material for: Resolving the full spectrum of human genome variation using Linked-Reads
Source: Genome Res. 2019 Apr;29(4):635–45. doi: 10.1101/gr.234443.118 (PMC6442396; doi:10.1101/gr.234443.118)
Supplement: Supplemental Material [file supp_gr.234443.118_Supplemental_longranger-2.2.zip › longranger-2.2.tar.bz2!/lr2.2-public/lib/stan/cmdstan/stan/doc/api/html/dump_8hpp_source.html]

Stan: src/stan/io/dump.hpp Source File


|  |  |
| --- | --- |
|  | Stan  2.5.0  probability, sampling & optimization |


- Main Page
- Related Pages
- Namespaces
- Classes
- Files

- File List
- File Members

All Classes Namespaces Files Functions Variables Typedefs Enumerations Enumerator Friends Macros Pages

- src
- stan
- io

dump.hpp

Go to the documentation of this file.

1 #ifndef STAN\_\_IO\_\_DUMP\_HPP

2 #define STAN\_\_IO\_\_DUMP\_HPP

3

4 #include <cctype>

5 #include <iostream>

6 #include <limits>

7 #include <map>

8 #include <sstream>

9 #include <stdexcept>

10 #include <string>

11 #include <vector>

12

13 #include <boost/lexical\_cast.hpp>

14 #include <boost/throw\_exception.hpp>

15

16 #include <stan/io/var\_context.hpp>

17 #include <stan/math/matrix.hpp>

18 #include <stan/math/meta/index\_type.hpp>

19 #include <stan/math/matrix/meta/index\_type.hpp>

20

21 namespace stan {

22

23  namespace io {

24

25  namespace {

26  size\_t product(std::vector<size\_t> dims) {

27  size\_t y = 1U;

28  for (size\_t i = 0; i < dims.size(); ++i)

29  y \*= dims[i];

30  return y;

31  }

32  }

33

42  class dump\_writer {

43  private:

44  std::ostream& out\_;

45

46  // checks doesn't contain quote char

47  void write\_name\_equals(const std::string& name) {

48  for (size\_t i = 0; i < name.size(); ++i)

49  if (name.at(i) == '"')

50  BOOST\_THROW\_EXCEPTION(

51  std::invalid\_argument ("name can not contain quote char"));

52  out\_ << '"' << name << '"' << " <- " << '\n';

53  }

54

55

56  // adds period at end of output if necessary for double

57  void write\_val(const double& x) {

58  std::stringstream ss;

59  ss << x;

60  std::string s;

61  ss >> s;

62  for (std::string::iterator it = s.begin();

63  it < s.end();

64  ++it) {

65  if (\*it == '.' || \*it == 'e' || \*it == 'E') {

66  out\_ << s;

67  return;

68  }

69  }

70  out\_ << s << ".";

71  }

72

73  void write\_val(const unsigned long long int& n) {

74  out\_ << n;

75  }

76

77  void write\_val(const unsigned long int& n) {

78  out\_ << n;

79  }

80

81  void write\_val(const unsigned int& n) {

82  out\_ << n;

83  }

84

85  void write\_val(const unsigned short& n) {

86  out\_ << n;

87  }

88

89  void write\_val(const long long& n) {

90  out\_ << n;

91  }

92

93  void write\_val(const long& n) {

94  out\_ << n;

95  }

96

97  void write\_val(const int& n) {

98  out\_ << n;

99  }

100

101  void write\_val(const short& n) {

102  out\_ << n;

103  }

104

105  void write\_val(const char& n) {

106  out\_ << n;

107  }

108

109

110  template <typename T>

111  void write\_list(T xs) {

112  typedef typename stan::math::index\_type<T>::type idx\_t;

113  out\_ << "c(";

114  for (idx\_t i = 0; i < xs.size(); ++i) {

115  if (i > 0) out\_ << ", ";

116  write\_val(xs[i]);

117  }

118  out\_ << ")";

119  }

120

121  template <typename T>

122  void write\_structure(std::vector<T> xs,

123  std::vector<size\_t> dims) {

124  out\_ << "structure(";

125  write\_list(xs);

126  out\_ << ',' << '\n';

127  out\_ << ".Dim = ";

128  write\_list(dims);

129  out\_ << ")";

130  }

131

132

133  void dims(double /\*x\*/, std::vector<size\_t> /\*ds\*/) {

134  // no op

135  }

136

137  void dims(int /\*x\*/, std::vector<size\_t> /\*ds\*/) {

138  // no op

139  }

140

141  template <typename T>

142  void dims(Eigen::Matrix<T,Eigen::Dynamic,Eigen::Dynamic> m,

143  std::vector<size\_t> ds) {

144  ds.push\_back(m.rows());

145  ds.push\_back(m.cols());

146  }

147

148  template <typename T>

149  void dims(Eigen::Matrix<T,Eigen::Dynamic,1> v,

150  std::vector<size\_t> ds) {

151  ds.push\_back(v.size());

152  }

153

154  template <typename T>

155  void dims(Eigen::Matrix<T,1,Eigen::Dynamic> rv,

156  std::vector<size\_t> ds) {

157  ds.push\_back(rv.size());

158  }

159

160  template <typename T>

161  void dims(std::vector<T> x, std::vector<size\_t> ds) {

162  ds.push\_back(x.size());

163  if (x.size() > 0)

164  dims(x[0],ds);

165  }

166

167  template <typename T>

168  std::vector<size\_t> dims(T x) {

169  std::vector<size\_t> ds;

170  dims(x,ds);

171  return ds;

172  }

173

174  bool increment(const std::vector<size\_t>& dims,

175  std::vector<size\_t>& idx) {

176  for (size\_t i = 0; i < dims.size(); ++i) {

177  ++idx[i];

178  if (idx[i] < dims[i]) return true;

179  idx[i] = 0;

180  }

181  return false;

182  }

183

184  template <typename T>

185  void write\_stan\_val(const std::vector<T>& x,

186  const std::vector<size\_t>& idx,

187  const size\_t pos) {

188  size\_t next\_pos = pos + 1;

189  write\_stan\_val(x[idx[pos]],idx,next\_pos);

190  }

191  void write\_stan\_val(const std::vector<double>& x,

192  const std::vector<size\_t>& idx,

193  const size\_t pos) {

194  write\_val(x[idx[pos]]);

195  }

196  void write\_stan\_val(const std::vector<int>& x,

197  const std::vector<size\_t>& idx,

198  const size\_t pos) {

199  write\_val(x[idx[pos]]);

200  }

201  void write\_stan\_val(const Eigen::Matrix<double,Eigen::Dynamic,Eigen::Dynamic>& x,

202  const std::vector<size\_t>& idx,

203  const size\_t pos) {

204  size\_t next\_pos = pos + 1;

205  write\_val(x(idx[pos],idx[next\_pos]));

206  }

207  void write\_stan\_val(const Eigen::Matrix<double,1,Eigen::Dynamic>& x,

208  const std::vector<size\_t>& idx,

209  const size\_t pos) {

210  write\_val(x[idx[pos]]);

211  }

212  void write\_stan\_val(const Eigen::Matrix<double,Eigen::Dynamic,1>& x,

213  const std::vector<size\_t>& idx,

214  const size\_t pos) {

215  write\_val(x[idx[pos]]);

216  }

217

218

219  template <typename T>

220  void write\_stan(const std::vector<T>& x) {

221  std::vector<size\_t> dims = dims(x);

222  out\_ << "structure(c(";

223  std::vector<size\_t> idx(dims.size(),0U);

224  for (size\_t count = 0; true; ++count) {

225  if (count > 0) out\_ << ", ";

226  write\_stan\_val(x,idx);

227  if (!increment(dims,idx)) break;

228  }

229  out\_ << "), .Dim = ";

230  write\_list(dims);

231  out\_ << ")";

232  }

233  void write\_stan(const std::vector<double>& x) {

234  write\_list(x);

235  }

236  void write\_stan(const std::vector<int>& x) {

237  write\_list(x);

238  }

239  void write\_stan(double x) {

240  write\_val(x);

241  }

242  void write\_stan(int x) {

243  write\_val(x);

244  }

245  void write\_stan(const Eigen::Matrix<double,1,Eigen::Dynamic>& x) {

246  write\_list(x);

247  }

248  void write\_stan(const Eigen::Matrix<double,Eigen::Dynamic,1>& x) {

249  write\_list(x);

250  }

251  void write\_stan(const Eigen::Matrix<double,

252  Eigen::Dynamic,Eigen::Dynamic>& x) {

253  typedef Eigen::Matrix<double,Eigen::Dynamic,Eigen::Dynamic>::Index Index;

254  out\_ << "structure(c(";

255  std::vector<double> vals;

256  for (Index m = 0; m < x.cols(); ++m) {

257  for (Index n = 0; n < x.rows(); ++n) {

258  if (m > 0 || n > 0) out\_ << ", ";

259  write\_val(x(m,n));

260  }

261  }

262  out\_ << "), .Dim = c(";

263  out\_ << x.rows() << ", " << x.cols();

264  out\_ << "))";

265  }

266

267  public:

268

272  dump\_writer() : out\_(std::cout) { }

273

280  dump\_writer(std::ostream& out) : out\_(out) { }

281

285  ~dump\_writer() { }

286

305  template <typename T>

306  void write(const std::string& name,

307  const T& x) {

308  write\_name\_equals(name);

309  write\_stan(x);

310  }

311

321  template <typename T>

322  void dump\_structure(std::string /\*name\*/,

323  std::vector<size\_t> dims,

324  std::vector<T> xs) {

325  if (xs.size() != product(dims))

326  BOOST\_THROW\_EXCEPTION(

327  std::invalid\_argument ("xs.size() != product(dims)"));

328  write\_structure(xs,dims);

329  }

330

339  template <typename T>

340  void dump\_list(std::string name,

341  std::vector<T> xs) {

342  write\_name\_equals(name);

343  write\_list(xs);

344  }

345

354  template <typename T>

355  void dump\_var(std::string name,

356  T x) {

357  write\_name\_equals(name);

358  write\_val(x);

359  }

360

361  };

362

434  class dump\_reader {

435  private:

436  std::string buf\_;

437  std::string name\_;

438  std::vector<int> stack\_i\_;

439  std::vector<double> stack\_r\_;

440  std::vector<size\_t> dims\_;

441  std::istream& in\_;

442

443  bool scan\_single\_char(char c\_expected) {

444  int c = in\_.peek();

445  if (in\_.fail()) return false;

446  if (c != c\_expected)

447  return false;

448  char c\_skip;

449  in\_.get(c\_skip);

450  return true;

451  }

452

453  bool scan\_optional\_long() {

454  if (scan\_single\_char('l'))

455  return true;

456  else if (scan\_single\_char('L'))

457  return true;

458  else

459  return false;

460  }

461

462  bool scan\_char(char c\_expected) {

463  char c;

464  in\_ >> c;

465  if (in\_.fail()) return false;

466  if (c != c\_expected) {

467  in\_.putback(c);

468  return false;

469  }

470  return true;

471  }

472

473  bool scan\_name\_unquoted() {

474  char c;

475  in\_ >> c; //

476  if (in\_.fail()) return false;

477  if (!std::isalpha(c)) return false;

478  name\_.push\_back(c);

479  while (in\_.get(c)) { // get turns off auto space skip

480  if (std::isalpha(c) || std::isdigit(c) || c == '\_' || c == '.') {

481  name\_.push\_back(c);

482  } else {

483  in\_.putback(c);

484  return true;

485  }

486  }

487  return true; // but hit eos

488  }

489

490  bool scan\_name() {

491  if (scan\_char('"')) {

492  if (!scan\_name\_unquoted()) return false;

493  if (!scan\_char('"')) return false;

494  } else if (scan\_char('\'')) {

495  if (!scan\_name\_unquoted()) return false;

496  if (!scan\_char('\'')) return false;

497  } else {

498  if (!scan\_name\_unquoted()) return false;

499  }

500  return true;

501  }

502

503

504  bool scan\_chars(const char \*s, bool case\_sensitive = true) {

505  for (size\_t i = 0; s[i]; ++i) {

506  char c;

507  if (!(in\_ >> c)) {

508  for (size\_t j = 1; j < i; ++j)

509  in\_.putback(s[i-j]);

510  return false;

511  }

512  // all ASCII, so toupper is OK

513  if ((case\_sensitive && c != s[i])

514  || (!case\_sensitive && ::toupper(c) != ::toupper(s[i]))) {

515  in\_.putback(c);

516  for (size\_t j = 1; j < i; ++j)

517  in\_.putback(s[i-j]);

518  return false;

519  }

520  }

521  return true;

522  }

523

524  bool scan\_chars(std::string s, bool case\_sensitive = true) {

525  for (size\_t i = 0; i < s.size(); ++i) {

526  char c;

527  if (!(in\_ >> c)) {

528  for (size\_t j = 1; j < i; ++j)

529  in\_.putback(s[i-j]);

530  return false;

531  }

532  // all ASCII, so toupper is OK

533  if ((case\_sensitive && c != s[i])

534  || (!case\_sensitive && ::toupper(c) != ::toupper(s[i]))) {

535  in\_.putback(c);

536  for (size\_t j = 1; j < i; ++j)

537  in\_.putback(s[i-j]);

538  return false;

539  }

540  }

541  return true;

542  }

543

544  size\_t scan\_dim() {

545  char c;

546  buf\_.clear();

547  while (in\_.get(c)) {

548  if (std::isspace(c)) continue;

549  if (std::isdigit(c)) {

550  buf\_.push\_back(c);

551  } else {

552  in\_.putback(c);

553  break;

554  }

555  }

556  scan\_optional\_long();

557  size\_t d = 0;

558  try {

559  d = boost::lexical\_cast<size\_t>(buf\_);

560  }

561  catch ( const boost::bad\_lexical\_cast &exc ) {

562  std::string msg = "value " + buf\_ + " beyond array dimension range";

563  BOOST\_THROW\_EXCEPTION (std::invalid\_argument (msg));

564  }

565  return d;

566  }

567

568  int scan\_int() {

569  char c;

570  buf\_.clear();

571  while (in\_.get(c)) {

572  if (std::isspace(c)) continue;

573  if (std::isdigit(c)) {

574  buf\_.push\_back(c);

575  } else {

576  in\_.putback(c);

577  break;

578  }

579  }

580  return(get\_int());

581  }

582

583  int get\_int() {

584  int n = 0;

585  try {

586  n = boost::lexical\_cast<int>(buf\_);

587  }

588  catch ( const boost::bad\_lexical\_cast &exc ) {

589  std::string msg = "value " + buf\_ + " beyond int range";

590  BOOST\_THROW\_EXCEPTION (std::invalid\_argument (msg));

591  }

592  return n;

593  }

594

595  double scan\_double() {

596  double x = 0;

597  try {

598  x = boost::lexical\_cast<double>(buf\_);

599  }

600  catch ( const boost::bad\_lexical\_cast &exc ) {

601  std::string msg = "value " + buf\_ + " beyond numeric range";

602  BOOST\_THROW\_EXCEPTION (std::invalid\_argument (msg));

603  }

604  return x;

605  }

606

607

608

609  // scan number stores number or throws bad lexical cast exception

610  void scan\_number(bool negate\_val) {

611  // must take longest first!

612  if (scan\_chars("Inf")) {

613  scan\_chars("inity"); // read past if there

614  stack\_r\_.push\_back(negate\_val

615  ? -std::numeric\_limits<double>::infinity()

616  : std::numeric\_limits<double>::infinity());

617  return;

618  }

619  if (scan\_chars("NaN",false)) {

620  stack\_r\_.push\_back(std::numeric\_limits<double>::quiet\_NaN());

621  return;

622  }

623

624  char c;

625  bool is\_double = false;

626  buf\_.clear();

627  while (in\_.get(c)) {

628  if (std::isdigit(c)) { // before pre-scan || c == '-' || c == '+') {

629  buf\_.push\_back(c);

630  } else if (c == '.'

631  || c == 'e'

632  || c == 'E'

633  || c == '-'

634  || c == '+') {

635  is\_double = true;

636  buf\_.push\_back(c);

637  } else {

638  in\_.putback(c);

639  break;

640  }

641  }

642  if (!is\_double && stack\_r\_.size() == 0) {

643  int n = get\_int();

644  stack\_i\_.push\_back(negate\_val ? -n : n);

645  scan\_optional\_long();

646  } else {

647  for (size\_t j = 0; j < stack\_i\_.size(); ++j)

648  stack\_r\_.push\_back(static\_cast<double>(stack\_i\_[j]));

649  stack\_i\_.clear();

650  double x = scan\_double();

651  stack\_r\_.push\_back(negate\_val ? -x : x);

652  }

653  }

654

655  void scan\_number() {

656  char c;

657  while (in\_.get(c)) {

658  if (std::isspace(c)) continue;

659  in\_.putback(c);

660  break;

661  }

662  bool negate\_val = scan\_char('-');

663  if (!negate\_val) scan\_char('+'); // flush leading +

664  return scan\_number(negate\_val);

665  }

666

667

668  bool scan\_seq\_value() {

669  if (!scan\_char('(')) return false;

670  if (scan\_char(')')) {

671  dims\_.push\_back(0U);

672  return true;

673  }

674  scan\_number(); // first entry

675  while (scan\_char(',')) {

676  scan\_number();

677  }

678  dims\_.push\_back(stack\_r\_.size() + stack\_i\_.size());

679  return scan\_char(')');

680  }

681

682  bool scan\_struct\_value() {

683  if (!scan\_char('(')) return false;

684  if (scan\_char('c')) {

685  scan\_seq\_value();

686  } else {

687  int start = scan\_int();

688  if (!scan\_char(':'))

689  return false;

690  int end = scan\_int();

691  if (start <= end) {

692  for (int i = start; i <= end; ++i)

693  stack\_i\_.push\_back(i);

694  } else {

695  for (int i = start; i >= end; --i)

696  stack\_i\_.push\_back(i);

697  }

698  }

699  dims\_.clear();

700  if (!scan\_char(',')) return false;

701  if (!scan\_char('.')) return false;

702  if (!scan\_chars("Dim")) return false;

703  if (!scan\_char('=')) return false;

704  if (scan\_char('c')) {

705  if (!scan\_char('(')) return false;

706  size\_t dim = scan\_dim();

707  dims\_.push\_back(dim);

708  while (scan\_char(',')) {

709  dim = scan\_dim();

710  dims\_.push\_back(dim);

711  }

712  if (!scan\_char(')')) return false;

713  }

714  else {

715  size\_t start = scan\_dim();

716  if (!scan\_char(':'))

717  return false;

718  size\_t end = scan\_dim();

719  if (start < end) {

720  for (size\_t i = start; i <= end; ++i)

721  dims\_.push\_back(i);

722  } else {

723  for (size\_t i = start; i >= end; --i)

724  dims\_.push\_back(i);

725  }

726  }

727  if (!scan\_char(')')) return false;

728  return true;

729  }

730

731  bool scan\_value() {

732  if (scan\_char('c'))

733  return scan\_seq\_value();

734  if (scan\_chars("structure"))

735  return scan\_struct\_value();

736  scan\_number();

737  if (!scan\_char(':'))

738  return true;

739  if (stack\_i\_.size() != 1)

740  return false;

741  scan\_number();

742  if (stack\_i\_.size() != 2)

743  return false;

744  int start = stack\_i\_[0];

745  int end = stack\_i\_[1];

746  stack\_i\_.clear();

747  if (start <= end) {

748  for (int i = start; i <= end; ++i)

749  stack\_i\_.push\_back(i);

750  } else {

751  for (int i = start; i >= end; --i)

752  stack\_i\_.push\_back(i);

753  }

754  dims\_.push\_back(stack\_i\_.size());

755  return true;

756  }

757

761  void print() {

762  std::cout << "var name=|" << name\_ << "|" << std::endl;

763  std:: cout << "dims=(";

764  for (size\_t i = 0; i < dims\_.size(); ++i) {

765  if (i > 0)

766  std::cout << ",";

767  std::cout << dims\_[i];

768  }

769  std::cout << ")" << std::endl;

770  std::cout << "float stack:" << std::endl;

771  for (size\_t i = 0; i < stack\_r\_.size(); ++i)

772  std::cout << " [" << i << "] " << stack\_r\_[i] << std::endl;

773  std::cout << "int stack" << std::endl;

774  for (size\_t i = 0; i < stack\_i\_.size(); ++i)

775  std::cout << " [" << i << "] " << stack\_i\_[i] << std::endl;

776  }

777

778

779  public:

783  dump\_reader() : in\_(std::cin) { }

784

790  dump\_reader(std::istream& in) : in\_(in) { }

791

795  ~dump\_reader() { }

796

797

803  std::string name() {

804  return name\_;

805  }

806

813  std::vector<size\_t> dims() {

814  return dims\_;

815  }

816

824  bool is\_int() {

825  // return stack\_i\_.size() > 0;

826  return stack\_r\_.size() == 0;

827  }

828

835  std::vector<int> int\_values() {

836  return stack\_i\_;

837  }

838

846  std::vector<double> double\_values() {

847  return stack\_r\_;

848  }

849

858  bool next() {

859  stack\_r\_.clear();

860  stack\_i\_.clear();

861  dims\_.clear();

862  name\_.erase();

863  if (!scan\_name()) // set name

864  return false;

865  if (!scan\_char('<')) // set <-

866  return false;

867  if (!scan\_char('-'))

868  return false;

869  try {

870  bool okSyntax = scan\_value(); // set stack\_r\_, stack\_i\_, dims\_

871  if (!okSyntax) {

872  std::string msg = "syntax error";

873  BOOST\_THROW\_EXCEPTION (std::invalid\_argument (msg));

874  }

875  }

876  catch (const std::invalid\_argument &e) {

877  std::string msg = "data " + name\_ + " " + e.what();

878  BOOST\_THROW\_EXCEPTION (std::invalid\_argument (msg));

879  }

880  return true;

881  }

882

883  };

884

885

886

902  class dump : public stan::io::var\_context {

903  private:

904  std::map<std::string,

905  std::pair<std::vector<double>,

906  std::vector<size\_t> > > vars\_r\_;

907  std::map<std::string,

908  std::pair<std::vector<int>,

909  std::vector<size\_t> > > vars\_i\_;

910  std::vector<double> const empty\_vec\_r\_;

911  std::vector<int> const empty\_vec\_i\_;

912  std::vector<size\_t> const empty\_vec\_ui\_;

922  bool contains\_r\_only(const std::string& name) const {

923  return vars\_r\_.find(name) != vars\_r\_.end();

924  }

925  public:

926

934  dump(std::istream& in) {

935  dump\_reader reader(in);

936  while (reader.next()) {

937  if (reader.is\_int()) {

938  vars\_i\_[reader.name()]

939  = std::pair<std::vector<int>,

940  std::vector<size\_t> >(reader.int\_values(),

941  reader.dims());

942

943  } else {

944  vars\_r\_[reader.name()]

945  = std::pair<std::vector<double>,

946  std::vector<size\_t> >(reader.double\_values(),

947  reader.dims());

948  }

949  }

950  }

951

960  bool contains\_r(const std::string& name) const {

961  return contains\_r\_only(name) || contains\_i(name);

962  }

963

972  bool contains\_i(const std::string& name) const {

973  return vars\_i\_.find(name) != vars\_i\_.end();

974  }

975

983  std::vector<double> vals\_r(const std::string& name) const {

984  if (contains\_r\_only(name)) {

985  return (vars\_r\_.find(name)->second).first;

986  } else if (contains\_i(name)) {

987  std::vector<int> vec\_int = (vars\_i\_.find(name)->second).first;

988  std::vector<double> vec\_r(vec\_int.size());

989  for (size\_t ii = 0; ii < vec\_int.size(); ii++) {

990  vec\_r[ii] = vec\_int[ii];

991  }

992  return vec\_r;

993  }

994  return empty\_vec\_r\_;

995  }

996

1004  std::vector<size\_t> dims\_r(const std::string& name) const {

1005  if (contains\_r\_only(name)) {

1006  return (vars\_r\_.find(name)->second).second;

1007  } else if (contains\_i(name)) {

1008  return (vars\_i\_.find(name)->second).second;

1009  }

1010  return empty\_vec\_ui\_;

1011  }

1012

1020  std::vector<int> vals\_i(const std::string& name) const {

1021  if (contains\_i(name)) {

1022  return (vars\_i\_.find(name)->second).first;

1023  }

1024  return empty\_vec\_i\_;

1025  }

1026

1034  std::vector<size\_t> dims\_i(const std::string& name) const {

1035  if (contains\_i(name)) {

1036  return (vars\_i\_.find(name)->second).second;

1037  }

1038  return empty\_vec\_ui\_;

1039  }

1040

1047  virtual void names\_r(std::vector<std::string>& names) const {

1048  names.resize(0);

1049  for (std::map<std::string,

1050  std::pair<std::vector<double>,

1051  std::vector<size\_t> > >

1052  ::const\_iterator it = vars\_r\_.begin();

1053  it != vars\_r\_.end(); ++it)

1054  names.push\_back((\*it).first);

1055  }

1056

1063  virtual void names\_i(std::vector<std::string>& names) const {

1064  names.resize(0);

1065  for (std::map<std::string,

1066  std::pair<std::vector<int>,

1067  std::vector<size\_t> > >

1068  ::const\_iterator it = vars\_i\_.begin();

1069  it != vars\_i\_.end(); ++it)

1070  names.push\_back((\*it).first);

1071  }

1072

1080  bool remove(const std::string& name) {

1081  return (vars\_i\_.erase(name) > 0)

1082  || (vars\_r\_.erase(name) > 0);

1083  }

1084

1085  };

1086

1087  }

1088

1089 }

1090 #endif

stan::io::dump::dump

dump(std::istream &in)

Construct a dump object from the specified input stream.

**Definition:** dump.hpp:934

stan::io::dump\_writer::dump\_writer

dump\_writer()

Construct a dump writer writing to standard output.

**Definition:** dump.hpp:272

stan::io::dump\_writer::write

void write(const std::string &name, const T &x)

Write a variable with the specified name and value.

**Definition:** dump.hpp:306

stan::io::dump\_reader::~dump\_reader

~dump\_reader()

Destroy this reader.

**Definition:** dump.hpp:795

stan::io::dump\_writer::dump\_structure

void dump\_structure(std::string, std::vector< size\_t > dims, std::vector< T > xs)

Write a structure variable with the specified name, dimensions, and integer or double values...

**Definition:** dump.hpp:322

stan::io::dump\_writer::~dump\_writer

~dump\_writer()

Destroy this writer.

**Definition:** dump.hpp:285

stan::io::dump\_reader::is\_int

bool is\_int()

Checks if the last item read is integer.

**Definition:** dump.hpp:824

stan::io::dump\_writer

Writes data into the S-plus dump format.

**Definition:** dump.hpp:42

stan::io::dump::names\_r

virtual void names\_r(std::vector< std::string > &names) const

Return a list of the names of the floating point variables in the dump.

**Definition:** dump.hpp:1047

stan::io::dump::contains\_r

bool contains\_r(const std::string &name) const

Return true if this dump contains the specified variable name is defined.

**Definition:** dump.hpp:960

stan::io::dump\_reader::double\_values

std::vector< double > double\_values()

Returns the floating point values from the last item if the last item read contained floating point v...

**Definition:** dump.hpp:846

stan::io::dump::names\_i

virtual void names\_i(std::vector< std::string > &names) const

Return a list of the names of the integer variables in the dump.

**Definition:** dump.hpp:1063

stan::io::dump\_writer::dump\_list

void dump\_list(std::string name, std::vector< T > xs)

Write a list variable with the specified name and integer or double values.

**Definition:** dump.hpp:340

stan::math::index\_type

Primary template class for the metaprogram to compute the index type of a container.

**Definition:** index\_type.hpp:21

stan::io::reader

A stream-based reader for integer, scalar, vector, matrix and array data types, with Jacobian calcula...

**Definition:** reader.hpp:34

stan::io::dump\_writer::dump\_var

void dump\_var(std::string name, T x)

Write a list variable with the specified name and integer or double values.

**Definition:** dump.hpp:355

var\_context.hpp

stan::math::dims

void dims(const T &x, std::vector< int > &result)

**Definition:** dims.hpp:13

stan::io::dump::contains\_i

bool contains\_i(const std::string &name) const

Return true if this dump contains an integer valued array with the specified name.

**Definition:** dump.hpp:972

stan::io::var\_context

A var\_reader reads array variables of integer and floating point type by name and dimension...

**Definition:** var\_context.hpp:29

stan::io::dump::dims\_r

std::vector< size\_t > dims\_r(const std::string &name) const

Return the dimensions for the double variable with the specified name.

**Definition:** dump.hpp:1004

index\_type.hpp

stan::io::dump\_reader::int\_values

std::vector< int > int\_values()

Returns the integer values from the last item if the last item read was an integer and the empty vect...

**Definition:** dump.hpp:835

stan::io::dump::vals\_r

std::vector< double > vals\_r(const std::string &name) const

Return the double values for the variable with the specified name or null.

**Definition:** dump.hpp:983

stan::math::e

double e()

Return the base of the natural logarithm.

**Definition:** constants.hpp:86

stan::io::dump\_writer::dump\_writer

dump\_writer(std::ostream &out)

Construct a dump writer writing to the specified output stream.

**Definition:** dump.hpp:280

stan::io::dump\_reader::name

std::string name()

Return the name of the most recently read variable.

**Definition:** dump.hpp:803

stan::io::dump\_reader::dims

std::vector< size\_t > dims()

Return the dimensions of the most recently read variable.

**Definition:** dump.hpp:813

stan::io::dump::vals\_i

std::vector< int > vals\_i(const std::string &name) const

Return the integer values for the variable with the specified name.

**Definition:** dump.hpp:1020

stan::io::dump\_reader::dump\_reader

dump\_reader()

Construct a reader for standard input.

**Definition:** dump.hpp:783

stan::io::dump\_reader

Reads data from S-plus dump format.

**Definition:** dump.hpp:434

index\_type.hpp

stan::io::dump

Represents named arrays with dimensions.

**Definition:** dump.hpp:902

stan::io::dump\_reader::next

bool next()

Read the next value from the input stream, returning true if successful and false if no further input...

**Definition:** dump.hpp:858

stan::io::dump\_reader::dump\_reader

dump\_reader(std::istream &in)

Construct a reader for the specified input stream.

**Definition:** dump.hpp:790

stan::io::dump::dims\_i

std::vector< size\_t > dims\_i(const std::string &name) const

Return the dimensions for the integer variable with the specified name.

**Definition:** dump.hpp:1034

matrix.hpp


---

[ Stan Home Page ]

*© 2011–2014,
Stan Development Team.*
